# L-Serine Treatment is Associated with Improvements in Behavior, EEG, and Seizure Frequency in Individuals with GRIN-Related Disorders Due to Null Variants

**DOI:** 10.1007/s13311-021-01173-9

**Published:** 2022-01-07

**Authors:** Ilona Krey, Sarah von Spiczak, Kathrine M. Johannesen, Christiane Hikel, Gerhard Kurlemann, Hiltrud Muhle, Diane Beysen, Tobias Dietel, Rikke S. Møller, Johannes R. Lemke, Steffen Syrbe

**Affiliations:** 1 Institute of Human Genetics, University of Leipzig Medical Center, Philipp-Rosenthal-Straße 55, 04103 Leipzig, Germany; 2 DRK-Northern German Epilepsy Center for Children and Adolescents, Schwentinental, Germany; 3 Department of Neuropediatrics, University Medical Center Schleswig-Holstein, Campus Kiel, Kiel, Germany; 4 Department of Epilepsy Genetics and Personalized Medicine, The Danish Epilepsy Centre, Dianalund, Denmark; 5 Department of Regional Health Research, University of Southern Denmark, Odense, Denmark; 6 Department of Children and Adolescents, GFO Kliniken Niederrhein, Dinslaken, Germany; 7 Hospital for Children, Bonifatius Hospital Lingen, Lingen, Germany; 8 Department of Paediatric Neurology, Antwerp University Hospital, Antwerp, Belgium; 9 Epilepsy Center Kork, Medical Faculty of the University of Freiburg, Kehl, Germany; 10 Center for Rare Diseases, University of Leipzig Medical Center, Leipzig, Germany; 11 Division of Paediatric Epileptology, Centre for Paediatrics and Adolescent Medicine, University Hospital Heidelberg, Heidelberg, Germany

**Keywords:** Developmental and epileptic encephalopathy, Epilepsy, Precision medicine, Targeted treatment, Retrospective observational case series

## Abstract

Pathogenic missense variants in *GRIN2A* and *GRIN2B* may result in gain or loss of function (GoF/LoF) of the N-methyl-D-aspartate receptor (NMDAR). This observation gave rise to the hypothesis of successfully treating GRIN-related disorders due to LoF variants with co-agonists of the NMDAR. In this respect, we describe a retrospectively collected series of ten individuals with *GRIN2A*- or *GRIN2B*-related disorders who were treated with L-serine, each within an independent *n-of-1* trial. Our cohort comprises one individual with a LoF missense variant with clinical improvements confirming the above hypothesis and replicating a previous *n-of-1* trial. A second individual with a GoF missense variant was erroneously treated with L-serine and experienced immediate temporary behavioral deterioration further supporting the supposed functional pathomechanism. Eight additional individuals with null variants (that had been interpreted as *loss-of-function* variants despite not being missense) again showed clinical improvements. Among all nine individuals with LoF missense or null variants, L-serine treatment was associated with improvements in behavior in eight (89%), in development in four (44%), and/or in EEG or seizure frequency in four (44%). None of these nine individuals experienced side effects or adverse findings in the context of L-serine treatment. In summary, we describe the first evidence that L-serine treatment may not only be associated with clinical improvements in GRIN-related disorders due to LoF missense but particularly also null variants.

**Supplementary Information:**

The online version contains supplementary material available at 10.1007/s13311-021-01173-9.

## Introduction

GRIN-related disorders comprise a spectrum of neurodevelopmental disorders related to pathogenic variants in one of the four currently known disease-associated GRIN genes, *GRIN1*, *GRIN2A*, *GRIN2B*, and *GRIN2D* – all encoding subunits of the N-methyl-D-aspartate receptor (NMDAR) [1–4]. Whereas *GRIN2A* and *GRIN2B* appear to be vulnerable to both missense and null variants [1, 2], heterozygous pathogenic variants in *GRIN1* or *GRIN2D* currently solely comprise missense [5]. In line with this observation, carriers of *GRIN2A* or *GRIN2B* null variants tend to display a milder phenotypic spectrum compared to carriers of missense variants [1, 2].

Functional studies have shown that pathogenic missense variants can result in a gain or loss of NMDAR function or in mixed and more complex functional consequences [6]. This observation enabled precision medicine approaches leading to the application of antagonists or agonists of the NMDAR, depending on the presence of a molecularly confirmed gain or loss of NMDAR function [1, 7–9].

In the absence of clinical trials, treatment reports remain anecdotal and often represent *n-of-1* trials [1, 7–9]. Similar to other observational studies of precision medicine approaches in genetic disorders, such as quinidine treatment in *KCNT1*-related epilepsy [10], these *n-of-1* trials have inspired treatment of several individuals with GRIN-related disorders. However, systematic analysis of treatment schemes and outcome parameters has not been reported so far.

Within our international network of collaborators, we have collected standardized information from individuals with GRIN-related disorders that had undergone targeted treatment with the orally available non-essential amino acid L-serine, which mediates co-agonistic effects on the NMDAR in neurons via its enantiomer D-serine [11]. In this retrospective, multicenter observational data collection, we report the genetic, clinical, and treatment data of nine individuals and analyze safety and efficacy of the individual treatments with L-serine.

## Materials and Methods

We retrospectively collected data on individuals with GRIN-related disorders that have undergone treatment with L-serine. Mandatory prerequisite for diagnosis of a GRIN-related disorder was the presence of a confirmed pathogenic variant in a GRIN gene, i.e., *GRIN2A* (NM_000833.5) or *GRIN2B* (NM_000834.3), according to the guidelines of the American College of Medical Genetics and Genomics [12]. Ten individuals from Germany *(n* = 8), Denmark (*n* = 1), and Belgium (*n* = 1) were identified and met our inclusion criteria. All respective individuals had been treated with L-serine and the treatment scheme resembled the publication of Soto et al. [8].

We collected genetic, clinical, and treatment data using a standardized questionnaire. Seizures and epilepsy were classified according to the latest ILAE (International League Against Epilepsy) definitions [13].

Successful treatment was often defined based on the clinician’s subjective impression or subjective report of parents/care givers. Efficacy was rated in four different categories that included behavior, cognitive/global development, EEG (electroencephalography) pattern, and seizure frequency. Different neurodevelopmental tests were performed in 8/10 cases, including Snijders-Oomen non-verbale intelligence test (SON-R) in three individuals, the Bayley Scales of Infant Development Third Edition (Bayley-III) [14] in two individuals, and the Vineland Adaptative Behavior Scales II (VABS-II) [15], the gross motor function test (GMFM-88) [16], the Canadian Occupational Performance Measure interview (COPM) [17], the Depressive Cognitions Scale (DCS) [18], the parent report screening assessment for autism spectrum disorders (social communication questionnaire, SCQ) [19], the test for reception of grammar (TROG-D) [20], and the Wechsler Preschool and Primary Scale of Intelligence – Fourth Edition (wppsi-IV) [21] in one case, each.

Thus, our report represents an unblinded, retrospective, observational multicenter analysis, not meeting the standards of a randomized controlled trial. This retrospective data collection has been approved by the ethics committee of the University of Leipzig (224/16-ek and 379/21-ek). Informed and voluntary consent according to the European General Data Protection Regulation and the Declaration of Helsinki has been obtained from each subject or legal guardian prior to recruitment and investigation.

## Results

Following the initial description of targeted treatment with L-serine in an individual with *GRIN2B*-related disorder due to a pathogenic loss-of-function missense variant in the context of an *n-of-1* trial [8], ten European individuals with GRIN-related neurodevelopmental disorders that received L-serine were identified. The spectrum of pathogenic variants of these ten affected individuals comprised:

- 1 *GRIN2B* loss-of-function (LoF) missense variant
- 1 *GRIN2B* gain-of-function (GoF) missense variant
- 5 *GRIN2B* null variants (two frameshift variants, two truncations, one splice variant)
- 3 *GRIN2A* null variants (one frameshift variant, one multi-exon deletion, one truncation)

In addition to the initial index case (#1) reported by Soto et al. [8], we present clinical and genetic data from nine novel individuals (#3–11) and one recently published case (#2) [22] in Table 1. Individuals were between 4 and 15 years of age. With the exception of individual #3 carrying a *GRIN2B* GoF missense variant, L-serine treatment lasted between 0.5 and > 2 years. Treatment is ongoing in 7/10 cases (#2, 4–8, 11). Individual treatment with L-serine was started independent of the report by Soto et al. [8] in 3/10 individuals (#2, 9, 10) explaining different protocols and dosages (see supplementary Table 1).

**Table 1:** Genetic information, clinical description, and treatment outcome of 11 individuals with L-serine supplementation

Individual	#1	#2	#3	#4	#5	#6	#7	#8	#9	#10	#11
Year of birth	2014	2014	2016	2009	2016	2015	2014	2014	2007	2005	2014
Sex	f	m	f	m	f	f	f	f	f	m	f
Gene	*GRIN2B*	*GRIN2B*	*GRIN2B*	*GRIN2B*	*GRIN2B*	*GRIN2B*	*GRIN2B*	*GRIN2B*	*GRIN2A*	*GRIN2A*	*GRIN2A*
Variant c	1657C > A	2065G > A	2087G > A	2539C > T	1016delT	2355delA	1368C > A	c.411 + 1G > T	176_179dupAGGC	Del Ex 12–14	2384G > A
Variant p	Pro553Thr	Gly689Ser	Arg696His	Arg847*	Leu339Argfs*25	Asp786Metfs*24	Cys456*	p.?	Ala61Glyfs*78	-	Trp795*
Variant type	missense	missense	missense	null	null	null	null	null	null	null	null
Functional Consequences	LoF	LoF	GoF	n.t	n.t	n.t	n.t	n.t	n.t	n.t	n.t
Origin	de novo	de novo	de novo	de novo	de novo	de novo	de novo	de novo	de novo	maternal	paternal
Ongoing or past epilepsy	No	Yes	No	Yes	No	No	Yes	No	Yes	Yes	Yes
EEG abnormalities	n.a	Focal slowing, sharp-waves	Bioccipital slowing	Gen. spike-waves, sharp-slow-waves	CSWS	Focal spikes	Gen. sharp-wave	Spike-wave, sharp-slow-waves	CSWS	CSWS, ESES	CSWS, ESES
Severity of DD/ID (IQ)	Severe	Severe	Moderate	Moderate	Mild	Mild	Mild	Mild	Normal (> 70)	Mild	Mild
Behavioral abnormalities	No	No	ASD, reduced anxiety	Aggressive	No	ASD, aggressive	ASD	Aggressive	Aggressive	Hyperactive	No
Muscular hypotonia	Yes	Yes	Yes	Yes	Yes	Yes	Yes	No	No	No	Yes
Movement disorder	Stereotypia	Dyskinesia	Paresis	Stereotypia	No	No	No	No	No	Oral dyspraxia	No
Other phenotypes	Sleep disturbance	No	No	No	No	No	No	No	Language disorder, psychiatric disorder, hypersalivation	Language disorder, psychiatric disorder, hypersalivation	No
Age at beginning of L-serine treatment	5 y 10 mo	3 y 2 mo	4 y	10 y	4 y	4 y 3 mo	6 y 5 mo	6 y 10 mo	12 y	14 y	6 y 3 mo
Duration of L-serine treatment	17 mo, ongoing	2 y, ongoing	2 d	2 y, ongoing	6 mo, ongoing	1 y, ongoing	7 mo, ongoing	12 mo, ongoing	7 mo; 4 mo stop; 12 mo	12 mo	3,5 mo, ongoing
L-serine dose (mg/kg/d)	500	600	500	500	560	500	500	500	100; 500	125	500; 850
AEDs used before L-serine treatment	VPA, LEV	VPA, LEV, CBD	no	LTG, VPA	STM	LEV	LEV	VPA, ESM, STM	STM, LEV	OXC, VPA, STM, CLB, MEM, PB, LEV	VPA
L-serine co-medication with other AED	n.a	PCM	no	VPA	STM	LEV	LEV	STM	no	PB + LEV	VPA
**Improvement of behavior**	**Yes**	**Yes**	**No**	**Yes**	**No**	**Yes**	**Yes**	**Yes**	**Yes**	**Yes**	**Yes**
**Improvement of DD/ID**	**Yes**	**Yes**	**No**	**Yes**	**Yes**	**No**	**No**	**Yes**	**No**	**No**	**No**
**Improvement of EEG**	**Yes**	**No**	**No**	**Yes**	**No**	**Yes**	**No**	**No**	**Yes**	**No**	**No**
**Improvement of sz**	**n.a**	**No**	**n.a**	**no**	**n.a**	**n.a**	**No**	**n.a**	**No**	**No**	**Yes**

*AED* anti-epileptic drug, *ASD* autism spectrum disorder, *CBD* cannabidiol, *CLB* clobazam, *CSWS* continuous spikes and waves during sleep, *d* day, *DD/ID* developmental delay/intellectual disability, *EEG* electroencephalography, *ESES* electrical status epilepticus in sleep, *ESM* ethosuximide, *f* female, *gen.* generalized, *GoF* gain of function, *IQ* intelligence quotient, *LEV* levetiracetam, *LoF* loss of function, *LTG* lamotrigine, *m* male, *MEM* memantine, *mo* month, *n.a.* not applicable, *n.t.* not testet, *OXC* oxcarbazepine, *PB* phenobarbital, *STM* sulthiame, *sz* seizure, *VPA* valproic acid, *y* year

L-serine was given as amino acid formula or encapsulated in all individuals. Dosages of L-serine ranged from 100 to 850 mg/kg body weight daily, usually divided in 3–4 dosages per day. All but one individual had concomitant anti-seizure medication (see Table 1).

Similar to the previously published index case [8] (#1), individual #2 carried a pathogenic *GRIN2B* LoF missense variant. Under L-serine, this individual experienced improvements in behavior and development and thus partially replicated the promising course of the published index case [8] (for details, see supplementary Table 1). L-serine treatment started in 2019 even before the initial publication [8] and is still ongoing.

In contrast to #1 and #2, individual #3 carried a pathogenic *GRIN2B* missense variant known to result in a GoF effect [6]. Immediately after the first application of L-serine, which was given wrongly in the context of a GoF variant, this individual experienced marked behavioral deterioration (for details see supplementary Table 1), which led to instant withdrawal of L-serine on the second day of application followed by *restitutio ad integrum* within hours.

Individuals #4 to #11 each carried a pathogenic null variant in either *GRIN2A* (#9–11) or *GRIN2B* (#4–8). None of which had previously been functionally tested. Under L-serine treatment, behavioral improvement was reported in eight of these nine individuals (89%). Four of nine individuals (44%) showed improvements in development and four (44%) on epileptiform activity in EEG and/or seizure frequency. According to available data of 6 individuals (#2, 4, 5, 9–11), improvements following L-serine treatment manifested within a few weeks to few months.

Improved alertness and vigilance were the most frequently observed behavioral improvements under L-serine treatment, reported in five of eight individuals (#2, 4, 6, 7, 10). Further behavioral changes comprised decrease of aggressive behavior (#4), improved concentration (#4, 8, 11), improved eye contact (#7), and less fearful behavior (#7). In one individual, the frequency of acoustic hallucinations was reduced (#9), and in individual #11, sleeping problems diminished. In two individuals (#9, 10), hypersalivation decreased. Assessment of treatment was difficult in #10 because of low-dose L-serine application and reduced adherence to treatment.

Improvements in development were reported in individuals #2, 4, 5, and 8, which was further objectified by an increase from 26 to 33% in the GMFM-88 in individual #2 and several improvements (e.g., 58 to 70 in working memory; 73 to 83 in vocabulary acquisition) in the wppsi-IV in individual #8. Additionally, individual #4 experienced mild and #5 substantial developmental and language improvements, which were observed by caring child neurologists and care givers, but were not objectified in standard tests.

Epileptiform potentials on EEG were present in all nine individuals with pathogenic LoF missense or null variants, and clinical seizures were observed in five individuals with null variants. Three of nine individuals (33%) had EEG improvements after initiation of L-serine treatment. These comprised a complete reduction of epileptic activity (#4), a reduction of sleep evoked epileptiform potentials (#6), and a decrease of the spike-wave index of continuous-spikes and waves during sleep in (#9). While EEG remained unchanged during treatment, individual #11 achieved seizure freedom under L-serine treatment. With the exception of individual #3 (with erroneously application of L-serine in the context of a *GRIN2B* GoF missense variant), none of the individuals experienced worsening of behavioral aspects, EEG patterns, seizure frequency, or other decline. Also, none of these individuals was reported to have experienced side effects or adverse findings in the context of L-serine treatment. Genetic details and phenotypic and treatment details are in Table 1; detailed information are displayed in Supplementary Table 1.

## Discussion

By reporting on ten individuals that had been treated independently as individual treatment trials with L-serine, we here present the most comprehensive observational retrospective data collection on agonist therapy of GRIN-related neurodevelopmental disorders allowing for several conclusions.

First, the response to L-serine treatment in individual #2 with pathogenic *GRIN2B* LoF missense variant appears to partially replicate the improvements of the initially published index case [8]. In contrast to Soto et al. [8], the frequency of epileptiform potentials on EEG did not significantly change in individual #2. However, motor development and particularly the patient’s behavior improved considerably.

Second, L-serine treatment in the context of a pathogenic *GRIN2B* GoF missense variant was associated with rapid behavioral deterioration in individual #3 followed by prompt and complete reversal after immediate stop of L-serine application. This observation not only illustrates the importance of functional confirmation of a loss of NMDAR function prior to application of NMDAR co-agonists. The fact that a co-agonist leads to deterioration in the presence of a gain of NMDAR function additionally supports the overall concept of therapeutically targeting the NMDAR receptor in GRIN-related disorders depending on the variant leading to a gain or loss of NMDAR function. Non-surprisingly, it suggests that co-agonists should be avoided in the context of NMDAR GoF variants.

Third, L-serine treatment appears to be beneficial also in the context of *GRIN2A* and *GRIN2B* null variants, as seen in individuals #4 to #11. The reason for the relatively larger number of such cases in this subcohort is likely due to the ambiguity of the term *loss-of-function variant.* Whereas this term is frequently used on DNA level to describe null variants, it also refers to missense variants resulting in a reduced protein function, commonly seen in ion channels, such as the NMDAR. Whereas Soto et al. [8] clearly referred to the latter missense variants, a misinterpretation for null variants in clinical practice could not be prevented. Nevertheless, carriers of *GRIN2A* and *GRIN2B* null variants presumably leading to haploinsufficiency appear to benefit from L-serine application as well – again particularly with respect to behavior.

Overall, beneficial effects of L-serine in all these individuals with null variants appeared to be mild.

Particularly, improvements in motor development as well as EEG pattern that have each been observed in a fraction of cases in our cohort may also (at least partially) have occurred within the normal course of an individual’s GRIN-related disorder. However, beneficial effects on behavior appear to be more pronounced and usually occurred within weeks after application. Furthermore, the distinctive response of individual #9 showed initial improvements with respect to EEG abnormalities as well as to psychiatric features after installation of L-serine treatment and a worsening of EEG as well as of speech and expressive language after its withdrawal. This supports that at least parts of the beneficial treatment responses observed in behavior, development, and EEG within our cohort are in fact L-serine-induced.

The size of our cohort was too small to evaluate differences in degree or spectrum of beneficial effects after L-serine application of carriers of LoF missense variants versus null variants in *GRIN2A* and *GRIN2B*. It also remains highly speculative whether the slightly different degree and spectrum of beneficial effects in the two cases with LoF missense variants (individual #2 with p.(Gly689Ser) located in S2 ligand binding site and index #1 from Soto et al. [8] with p.(Pro553Thr) located in the M1 transmembrane domain) were related to the location of the individual variant and the distinct type of loss of NMDAR function.

Given the non-standardized individual *n-of-1* trials from different European centers, our case series has several limitations that need to be considered when evaluating our results; these include a small group with different genotypes, no standardized dosing regimen, lack of blood or plasma levels of serine and anti-epileptic drugs, the lack of a control group, and non-standardized and partially subjective outcome parameters. In addition, one has to consider that functional consequences from genetic variants were not tested and were mostly concluded from a putative genetic effect (i.e., null variants leading to haploinsufficiency).

Despite these limitations, we were able to report several important and useful observations that will help in planning and conducting further clinical trials.

Regarding safety, treatment with L-serine, even in dosages up to 850 mg/kg body weight per day, was well tolerated in all subjects, and no adverse drug events were reported during the observation period. Regarding efficacy and effects on epilepsy and development, several findings can be delineated from our series.

In conclusion, this retrospective observational data collection argues for a good safety profile of L-serine in children with genetic disorders due to NMDAR dysfunction. Given the desperate situation and frequent resistance to standard anti-seizure medication, novel treatments are urgently needed. Our data will help to plan future trials addressing questions on genotype specific effects, dose recommendations, and effect on epilepsy and neurodevelopment. Therefore, we recommend a larger controlled clinical trial differentiating between LoF missense and null variants in the different GRIN genes according to our most detailed and precise testing scheme of individual #2 before, during and after treatment with L-serine. Such trials should also include measurement of plasma levels of serine and the other anti-epileptic drugs. Furthermore, our observations provide evidence that application of L-serine may worsen the clinical situation in individuals carrying GoF variants and thus should only be considered in the context of proven LoF missense or null variants.

## Supplementary Information

Below is the link to the electronic supplementary material.

Supplementary file1 (XLSX 70.2 KB)

Supplementary file2 (PDF 464 KB)

Supplementary file3 (PDF 465 KB)

Supplementary file4 (PDF 466 KB)

Supplementary file5 (PDF 465 KB)

Supplementary file6 (PDF 731 KB)

Supplementary file7 (PDF 465 KB)

Supplementary file8 (PDF 466 KB)

Supplementary file9 (PDF 465 KB)

Supplementary file10 (PDF 466 KB)

Supplementary file11 (PDF 467 KB)

Supplementary file12 (PDF 465 KB)
